# Two extraction-free reverse transcription loop-mediated isothermal amplification assays for detection of SARS-CoV-2

**DOI:** 10.1186/s12879-021-06876-0

**Published:** 2021-11-17

**Authors:** Meng Yee Lai, Fatma Diyana Mohd Bukhari, Nur Zulaikha Zulkefli, Ilyiana Ismail, Nur Izati Mustapa, Tuan Suhaila Tuan Soh, Afifah Haji Hassan, Kalaiarasu M. Peariasamy, Yee Leng Lee, Jeyanthi Suppiah, Ravindran Thayan, Yee Ling Lau

**Affiliations:** 1 Department of Parasitology, Faculty of Medicine, University of Malaya, 50603 Kuala Lumpur, Malaysia; 2 Department of Pathology, Hospital Sungai Buloh, Ministry of Health, Kuala Lumpur, Malaysia; 3 Institute for Clinical Research, National Institutes of Health, Ministry of Health, Kuala Lumpur, Malaysia; 4 Clinical Research Centre, Hospital Sungai Buloh, Ministry of Health, Kuala Lumpur, Malaysia; 5 Virology Unit, Infectious Disease Research Centre, Institute for Medical Research, National Institutes of Health, Ministry of Health, Kuala Lumpur, Malaysia

**Keywords:** SARS-CoV-2, Isothermal detection, Coronaviruses, Rapid diagnosis, RT-LAMP

## Abstract

**Background:**

Current assays for detection of severe acute respiratory syndrome coronavirus 2 (SARS-CoV-2) rely on time consuming, costly and laboratory based methods for virus isolation, purification and removing inhibitors. To address this limitation, we propose a simple method for testing RNA from nasopharyngeal swab samples that bypasses the RNA purification step.

**Methods:**

In the current project, we have described two extraction-free reverse transcription loop-mediated isothermal amplification (RT-LAMP) assays for the detection of SARS-CoV-2 by using *E* gene and *RdRp* gene as the targets.

**Results:**

Here, results showed that reverse transcription loop-mediated isothermal amplification assays with 88.4% sensitive (95% CI: 74.9–96.1%) and 67.4% sensitive (95% CI: 51.5–80.9%) for *E* gene and *RdRp* gene, respectively.

**Conclusion:**

Without the need of RNA purification, our developed RT-LAMP assays for direct detection of SARS-CoV-2 from nasopharyngeal swab samples could be turned into alternatives to qRT-PCR for rapid screening.

**Supplementary Information:**

The online version contains supplementary material available at 10.1186/s12879-021-06876-0.

## Background

Coronavirus disease 2019 or COVID-19 is a contagious viral infection that attacks humans, primarily the throat and lungs. This disease, which is caused by severe acute respiratory syndrome coronavirus 2 (SARS-CoV-2), must infect living cells in order to reproduce. The presence of new COVID-19 variants makes the disease more infectious and rapidly emerging. In today’s scenario where the world is struggling and fighting against the novel coronavirus, real-time polymerase chain reaction or RT-PCR is the most reliable test for the detection of COVID-19 infection [1–4]. However, looking at the reality of the current situation where the pandemic is spread so broadly, there is a need to be thinking about another scale of testing which is simple-to-use, fast and cost effective. Consequently, isothermal methods such as loop-mediated isothermal amplification (LAMP) has come to the fore as a great alternative to the RT-PCR method [5–7].

Previously, laboratory screening test of SARS-CoV-2 using RT-PCR exploits a few different types of detection gene including envelope (*E*) gene, nucleocapside (*N*) gene [2, 8] and RNA dependent RNA Polymerase (*RdRp*) gene [2]. These genes are significant for overall function of coronavirus and conserved [9]. These genes have been used in RT-LAMP as well [5, 7–11], however most of these methods required RNA extraction and purification using commercial kits which is costly and time consuming. In this study, we amplified *E* gene and *RdRp* gene using RT-LAMP assay using direct nasopharyngeal swab specimens, without RNA extraction and purification. With this optimization, detection of SARS-CoV-2 could be performed directly on clinical samples as a point-of-care test utilizing readily available reagents and equipment.

## Methods and results

### Source of samples

A total of 43 rRT-PCR positive (C_t_ values range: 14.4–38.85) and 15 rRT-PCR negative nasopharyngeal and oropharyngeal swabs samples were involved in these RT-LAMP assays. During rRT-PCR assay, the RNA from these samples were extracted by using QIAamp viral RNA Mini kit (Qiagen, Hilden, Germany) according to the manufacturer’s instructions. These samples involved in this study were obtained from Hospital Sungai Buloh and Institute for Medical Research, Malaysia. Upon collection, each swab sample was placed into a sterile vial containing 2-mL of viral transport media (VTM) and preheated at 65 °C for 1 h. An aliquot of 250 μL of each swab samples were frozen and sent to University of Malaya on dry ice. This study was approved by UMMC Medical Ethics Committee (202041-8418) and Medical Research Ethics Committee (MREC) Ministry of Health Malaysia (NMRR-20-2344-56994).

### RNA extraction-free protocol

Here, we described a column-free RNA preparation method. The RNA was extracted from swabs samples using bovine serum albumin (BSA) based method following the procedures described by Plante et al. [12] and Wozniak et al. [13] with minor modification. A total of 2.5 µL swab sample in VTM was mixed with 47.5 µL of 1 mg/mL BSA solution (1:20 ratio). The mixture was then vortex for 30 s followed by quick spin. The supernatant (14.16 µL) was used as a template for RT-LAMP assay.

### RT-LAMP assays

*E* gene and *RdRp* gene of SARS-CoV-2 were selected as our target regions in the present study. Table 1 indicates the primer sequences of both genes. The primers were specifically designed using Primer-Explorer V4 software (Eiken Chemical Co., Ltd., Tokyo, Japan) based on SARS-CoV-2 envelope protein (GenBank accession no: MT192773.1, MT192772.1, MT669322.1 and MT646120.1) and RNA-dependent RNA Polymerase (GenBank accession no: LR757995.1, LR757998.1 and MT192773.1). Each of the samples was tested separately with 2 primers sets following BSA extraction step. The assay was performed in a total reaction volume of 30 μL consisting of 3 µL of 10 × isothermal amplification buffer II, 1.8 µL of 100 mM MgSO_4_, 1.68 µL of 100 mM dNTPs, 1.2 µL of *Bacillus stearothermophilus* (*Bst*) 3.0 DNA polymerase (NEB, Ipswich, United States), 4.56 µL of primer mix, 1.2 µL RNaseOUT™ recombinant ribonuclease inhibitor (Invitrogen, California, United States), 2.4 µL of hydroxynaphthol blue (HNB) (Sigma, St. Louis, USA) and 14.16 µL of RNA. The reaction mixture was incubated in Loopamp Real-Time Turbidimeter LA 500 (Eiken Chemical Co., Ltd., Taito-ku, Japan) at 65 °C for 45 min. Additional upfront of HNB in the master mix was used for direct observation of colour changes on end products. Positive LAMP reaction shows sky blue whereas negative reaction remained in violet colour. The cost per reaction of LAMP (~ $1.77 USD/reaction) is much cheaper than rRT-PCR (~ $11.94 USD/reaction).

**Table 1:** LAMP *E* gene and *RdRp* gene primers used in this study

Primer	Sequence (5′–3′)
*E* gene	
FIP	CGCAGTAAGGATGGCTAGTGTAGCGTACTTCTTTTTCTTGCTT
BIP	TCGATTGTGTGCGTACTGCTGTTTTTAACACGAGAGTAAACGT
FLP	CTAGCAAGAATACCACG
BLP	GTTAACGTGAGTCTTG
F3	TTTCGGAAGAGACAGGTAC
B3	AGGAACTCTAGAAGAATTCAGA
*RdRp* gene	
FIP	TGAGCACACTCATTAGCTAATCTCGCAAACATACAACGTGTTG
BIP	GAGTGAAATGGTCATGTGTGGAAGCAGTTGTGGCATCTC
FLP	GAAACGGTGTGACAAGCT
BLP	GTTAAACCAGGTGGAACCTC
F3	TGGCCTCACTTGTTCTTG
B3	AGCTTGACAAATGTTAAAAACAC

### Analytical sensitivity and specificity test

To determine the analytical sensitivity for *E* gene and *RdRp* gene, a recombinant plasmid carrying *E* gene or *RdRp* fragments of SARS-CoV-2 were constructed. F3 and B3 primers set were used to amplify the target genes from synthetic fragments (Sangon Biotec Co., Ltd., Shanghai, China). The PCR conditions were as follows: denaturation at 94 °C for 5 min, 30 cycles of 94 °C for 30 s, 55 °C for 60 s and 72 °C for 30 min, and a final extension step at 72 °C for 10 min. Following the PCR amplification, the amplified products were purified prior to cloned into pGEM-T vector (Promega Corporation, Madison, USA) and transformed into TOP10F’ *E. coli* competent cells. The recombinant plasmids were extracted using Qiagen Spin Miniprep kit (Qiagen, Hilden, Germany) and sent for sequencing to confirm its identity. The pGEM-T vector containing either *E* gene or *RdRp* inserts were linearized by *Sal*I and transcribed into RNA by RiboMAX™ Large Scale RNA Production Systems (Promega Corporation, Madison, United States) according to the manufacturer’s instruction. The RNA copy number was calculated based on the following formula: copies/µL = [(6.02 × 10^23^ × 10^–9^) × concentration (ng/µL)]/(fragment length × 340)^4^). Ten-fold serial dilutions of the transcribed RNA ranging from 1 × 10^6^ copies/µL to 1 copy/µL were prepared. Each of the dilution factors was tested in triplicates in RT-LAMP assay. Results showed that the limit of detection of the transcribed RNA both *E* gene and *RdRp* gene was 1 copy/µL.

### Clinical sensitivity and specificity test

The specificity of the *E* gene and *RdRp* gene in RT-LAMP assay was tested using genomic RNA of coronavirus, adenovirus 4, influenza A H3N2, influenza B, novel influenza A H1N1, parainfluenza virus 1, parainfluenza virus 2, parainfluenza virus 3, respiratory syncytial virus (subtype A) and syncytial virus (subtype B) (Vircell, Granada, Spain). Each of the genomic RNA was tested in triplicates. Results showed that the specificity was 100%.

Next, the clinical sensitivity and specificity of RT-LAMP was evaluated using 58 swabs samples. The sensitivity was calculated based on the formula: (number of true positives)/(number of true positives + number of false negatives) and the specificity was calculated as (number of true negatives)/(number of true negatives + number of false positives). Out of 43 rRT-PCR positive samples and 15 rRT-PCR negative samples, RT-LAMP using *E* gene detected only 38 samples as positive and 13 samples as negative. The RT-LAMP using *E* gene was 88.4% sensitive (95% CI: 74.9–96.1%) and 86.7% specific (95% CI: 59.5–98.3). Positive and negative predictive values for RT-LAMP targeting *E* gene were 95.0% (95% CI: 83.88–98.58) and 72.2% (95% CI: 52.70–85.85), respectively. In other words, 95.0% of all swabs samples tested for the detection of COVID-19 infection were actually had the SARS-CoV-2 infections and 72.2% of swabs samples were truly virus-free infection tested by RT-LAMP using *E* gene. On the other hand, for RT-LAMP using *RdRp* gene, 29 rRT-PCR positive samples were detected as positive and 13 rRT-PCR negative samples were detected as negative. RT-LAMP using *RdRp* gene was 67.4% sensitive (95% CI: 51.5–80.9%) and 86.7% specific (95% CI: 59.5–98.3%) (Additional file 1: Table S1). The positive predictive value is 93.5% (95% CI: 79.69–98.17) and the negative predictive value is 48.1% (95% CI: 36.64–59.86).

## Discussion

To mitigate the COVID-19 outbreak, development of simple and rapid molecular based methods for direct detection of SARS-CoV-2 virus in respiratory samples is an urgent need. To bypass the RNA purification step, many alternative methods enabling simple nucleic acid extraction from SARS-CoV-2 have been described. Most of the investigators focused on RNA extraction from saliva samples instead of swab samples. Such methods involve using a commonly found reagent in molecular biology laboratories, such as bovine serum albumin (BSA), proteinase K and formamide. Lalli et al. managed to extract RNA from saliva samples by combination of heating steps and proteinase K treatment [14]. Result shows that the limit of detection (LOD) of RT-LAMP was down to 10^2^ viral genomes. Meanwhile, Li et al. reported an extraction-free RT-LAMP method by incorporation of 6% formamide into reaction mixture. They managed to obtain 5 copies/μL within 45 min [15]. Wei et al. reported a RT-LAMP based testing on RNA-spike samples by using an in-house lysis buffer. Results showed the LOD of this method as low as 2.5 copies/µL RNA [16]. Meanwhile, Dudley et al. developed a fluorescence-based RT-LAMP test using direct nasopharyngeal swab samples without any treatment and showed a LOD of 1 × 10^2^–1 × 10^4^ copies/µL [17]. Mautner et al. has described a RT-LAMP assay for the detection of SARS-CoV-2 directly from pharyngeal swab samples by using *ORFB* and *N* gene as the target regions [18]. Their report showed a LOD of 100 copies/µL RNA for both genes. In the work presented here, we developed a BSA-based extraction method for the detection of SARS-CoV-2. By adding 2.5 µL of VTM into 47.5 µL BSA (1 mg/mL) followed by 30 s vortex, the sample can be served directly as the template for RT-LAMP assay. We managed to develop two RT-LAMP assays for direct detection of SARS-CoV-2 in VTM in 45 min. RT-LAMP results showed that sensitivity for both *E* gene and *RdRp* was 1 copy/µL. These RT-LAMP assays are rapid and simple if compared to conventional nucleic acid extraction kits as no purification step is required. Overall RNA extraction method took approximately 2 min to complete.

In the two extraction-free assays developed here, clinical sensitivity of RT-LAMP based on *E* gene and *RdRp* gene were 88.4 and 67.4%, respectively. However, our results indicated a barely lower sensitivity of RT-LAMP assay than other studies. Nawattanapaiboon et al. obtained 95.8% sensitivity by using *RdRp* as target gene during their development of colorimetric RT-LAMP for SARS-CoV-2 detection [19]. Jang et al. managed to obtain 93.9% sensitivity of the *RdRp* gene in the development of multiplex RT-LAMP for on-site SARS-CoV-2 diagnosis [20]. Meanwhile for *E* gene as the target region in RT-LAMP assay, Yang et al. shows a 98.5% sensitivity during the development of triplex RT-LAMP assay for the detection of SARS-CoV-2 [21]. Pang et al. demonstrated a 94% sensitivity of *E* gene during their development of RT-LAMP combination with clustered regularly interspaced short palindromic repeats (CRISPRs) technology in the detection of SARS-CoV-2 [22]. All these assays were conducted using commercial kits. Our developed RT-LAMP presented a lower sensitivity as compared to other findings, it may need further optimization. On top of that, RNA maybe degraded during storage or shipment. Further study basing on larger sample size could have generated more accurate results.

Apart from this, we performed several trials of using unprocessed swab samples, at which the collected swab samples were not subjected to any pre-treatment with chemicals and heat-inactivated procedures. As suggested by Ladha et al. only 10% of the amount from total reaction volume was used as a template. Several other amounts of swab samples, 1, 2.5 and 5 µL were tested as well. Unfortunately, no amplification result was obtained [23]. According to Anahtar et al. direct RT-LAMP testing of unprocessed specimens from nasopharyngeal swab samples could only reliably detect samples with abundant SARS-CoV-2 (3 × 10^6^ copies/mL) [24].

## Conclusions

Here we have demonstrated a simple and inexpensive BSA based extraction method for RT-LAMP assays for the detection SARS-CoV-2. This assay can be performed in any clinical laboratory, diagnostic centres and mobile laboratory with a biological safety level cabinet, as it does not require any other specialized equipment. Taken together, this assay can be employed as an alternative diagnosis method in resource limited settings and thereby alleviating supply kits and reagents issues.

### Supplementary Information

**Additional file 1**: **Table S1**. RT-LAMP and real time RT-PCR results.

## Data Availability

The analysed data sets generated during the study are available from the corresponding author on reasonable request.

## Abbreviations

COVID-19: Coronavirus disease 2019
SARS-CoV-2: SARS-associated coronavirus
*E* gene: Envelope protein
RdRp gene: RNA-dependent RNA polymerase
