# Supplementary material for: Two extraction-free reverse transcription loop-mediated isothermal amplification assays for detection of SARS-CoV-2
Source: BMC Infect Dis. 2021 Nov 17;21:1162. doi: 10.1186/s12879-021-06876-0 (PMC8595270; doi:10.1186/s12879-021-06876-0)
Supplement: Supplementary file 1 — Additional file 1: Table S1. RT-LAMP and real time RT-PCR results. [file 12879_2021_6876_MOESM1_ESM.docx]

**Additional file 1**

**Table S1:** **RT-LAMP and real time RT-PCR results.**

| **Patient Sample No.** | **RT-LAMP** | | **RT-PCR (Ct-value)** |
| --- | --- | --- | --- |
|  | ***E* gene (min)** | ***RdRp* gene (min)** |  |
| N8585 | 25.30 | Neg^^[[1]](#endnote-1)^^ | 21.80 |
| N8586 | 27.24 | Neg | 19.72 |
| N8588 | Neg | Neg | 18.08 |
| N8589 | 30.48 | Neg | 18.36 |
| N8598 | 31.48 | 36.54 | 17.35 |
| N8669 | Neg | Neg | 25.25 |
| N8759 | 41.18 | Neg | 23.84 |
| N8829 | 32.36 | Neg | 15.51 |
| N8888 | 34.18 | Neg | 27.45 |
| 42889 | 20.00 | 34.00 | 18.37 |
| 42903 | 32.03 | 36.00 | 16.29 |
| 45107 | 25.00 | 27.48 | 14.38 |
| 355250 | 30.42 | 33.54 | 13.95 |
| 51716 | 29.06 | Neg | 22.13 |
| 43503 | 27.12 | 38.30 | 21.07 |
| 46759 | 28.24 | 37.12 | 15.85 |
| 25306 | 28.48 | 32.54 | 17.01 |
| 39298 | 28.00 | 32.06 | 21.47 |
| 65760 | 23.42 | 29.36 | 14.86 |
| 343120 | 23.48 | 36.24 | 15.82 |
| 342150 | 27.06 | 30.48 | 18.14 |
| 342149 | 27.24 | 32.42 | 23.33 |
| 342649 | 32.00 | 36.00 | 19.67 |
| 342652 | 25.24 | 37.18 | 21.68 |
| 342655 | 29.54 | 36.36 | 21.39 |
| 342660 | 27.42 | 28.36 | 24.44 |
| 342678 | 26.18 | 26.24 | 24.07 |
| 341923 | Neg | 30.36 | 15.18 |
| 342328 | 28.42 | 26.00 | 14.40 |
| N10556 | Neg | 35.24 | 23.19 |
| N10609 | 31.00 | 35.48 | 28.84 |
| N10606 | 31.24 | 33.48 | 23.71 |
| N10596 | 31.30 | 39.48 | 27.56 |
| N10540 | 32.12 | 36.54 | 18.26 |
| N10229/10299 | 21.00 | 38.48 | 23.22 |
| N10597 | 27.30 | 34.42 | 36.27 |
| N10463 | 32.12 | Neg | 35.19 |
| N10348 | 29.24 | Neg | 35.87 |
| N10241 | 26.30 | 30.12 | 36.06 |
| N10467 | 26.24 | Neg | 36.67 |
| N10184 | 32.54 | 37.30 | 38.85 |
| N10440A | 19.24 | Neg | 33.38 |
| N10376A | Neg | Neg | 34.90 |
| N10454 | Neg | Neg | Neg |
| N10455 | Neg | Neg | Neg |
| N10472 | 33.06^^[[2]](#endnote-2)^^ | Neg | Neg |
| N10481 | Neg | Neg | Neg |
| N10479 | Neg | Neg | Neg |
| N10422 | Neg | Neg | Neg |
| N10435 | Neg | Neg | Neg |
| N10441 | Neg | Neg | Neg |
| N10458 | Neg | Neg | Neg |
| N10459 | Neg | Neg | Neg |
| N8795A | Neg | Neg | Neg |
| 76247 | Neg | Neg | Neg |
| 76248 | Neg | Neg | Neg |
| 342136 | 28.24 | 29.00 | Neg |
| 342137 | Neg | 28.36 | Neg |

1. False negatives [↑](#endnote-ref-1)
2. False positives [↑](#endnote-ref-2)
